# A Neuronavigation-Assisted Endoscopic Ventriculocystocisternostomy of a Suprasellar Arachnoid Cyst: A 2D Video Case Presentation

**DOI:** 10.7759/cureus.14957

**Published:** 2021-05-11

**Authors:** Lekhaj C Daggubati, Faiza Boukerche, Elias Rizk

**Affiliations:** 1 Department of Neurosurgery, Penn State Health Milton S. Hershey Medical Center, Hershey, USA; 2 Department of Neurosurgery, Penn State College of Medicine, Hershey, USA

**Keywords:** suprasellar arachnoid cyst, ventriculocystocisternostomy, endoscopic cyst fenestration, intradural arachnoid cyst, ventriculocystostomy

## Abstract

*Background and Importance:* Suprasellar arachnoid cysts are insidious and progressive ventricular cysts that cause hydrocephalus, ataxia, optic, and endocrinological symptoms. These cysts can be successfully treated via an endoscopic ventriculocystocisterostomy (VCC).

*Clinical Presentation:* A three-year-old patient presented with emesis and bobble-head doll syndrome with a suprasellar arachnoid cyst. Through our video, we present our neuronavigation-assisted endoscopic VCC without cautery and the relevant neuroanatomy.

*Conclusion:* Neuroendoscopic fenestration is a preferred approach over resection or shunting for a suprasellar arachnoid cyst. In addition, VCC has decreased recurrence and increased radiologic improvement over a ventriculocystostomy (VC).

## Introduction

Suprasellar cysts are benign, possibly congenital, collections of cerebrospinal fluid whose pathophysiology is poorly understood. However, it is postulated that these primary cysts are due to unexplained splitting or tearing of the arachnoid membrane [1]. Secondary cysts, on the other hand, are due to trauma, surgery, or intracranial hemorrhage. The primary cysts start as an abnormality in the membrane of Liliequist or the interpeduncular cistern and expand over time, displacing the floor of the third ventricle, optic chiasm, mammillary bodies, and pituitary stalk [2, 3]. The cyst's histological characteristic is rarely analyzed and is mainly classified as an arachnoid cyst [1]. Furthermore, suprasellar cysts' frequency and occurrence is 1.4% in adults and 2.6% in children [4-6]. The treatment has evolved over time; currently, it consists of either resection, shunting, or fenestration of the cyst [7]. Technological advances have increased safety and allowed minimally invasive approaches. We present our case of a suprasellar cyst fenestration via a ventriculocystocisternostomy (VCC) without cautery.

## Case presentation

Our patient is a three-year-old male who presented with worsening ataxia, emesis, and a history of over nine months of interval bobble-head doll syndrome. He was initially misdiagnosed with tics. An MRI of the brain was performed due to the ataxia; and, a suprasellar cyst with obstructive hydrocephalus was found. In our VCC, we approached via the right lateral ventricular with the assistance of neuronavigation. The apical fenestration was made without cautery and continuous maneuvering of the apical surface without blunt instruments. Once inside the cyst, the neuroendoscope is carefully placed at the expanded third ventricle ventral floor to the clivus. Using the blunt neuronavigation probe, a cystocisternotomy was made slightly left eccentric at the floor. Using the eccentric approach, blunt instruments, and avoiding cautery, we minimized the risk to the neurovasculature (i.e., basilar artery). The patient had rapid resolution of his bobble-head doll syndrome by discharge on a postoperative day 3 (Video 1).

**Video 1:** An endoscopic ventriculocystocisternostomy of a suprasellar arachnoid cyst: A 2D video case presentation.

During the four-month post-surgical follow-up visit, the patient presented for a repeat MRI to assess the clinical and radiological progression. A contrast MRI demonstrated that the suprasellar cyst has decreased in size from 4.5 cm upon presentation to 2.7 cm at the most recent MRI. Furthermore, no parenchymal hemorrhage or edema was appreciated. Clinically, the patient’s neurological symptoms have resolved entirely and stable at his eight-month follow-up.

An Institutional Review Board/Ethics Committee approval was not sought out since this is a case report. Consent was attained from the patient's parents to allow the presentation of the case and was in compliance with CARE guidelines.

## Discussion

Suprasellar arachnoid cysts are uncommon ventricular pathology that necessitates surgical intervention due to obstructive hydrocephalus, gait ataxia, bobblehead doll movement, visual disturbances, and endocrine dysfunction [8]. The current treatment approaches include microsurgical fenestration via craniotomy, neuroendoscopy fenestration, and cytoperitoneal shunting [7]. The distorted neurovascular anatomy from the norm increases the perioperative risk. Our case shows a neuronavigation-assisted non-cautery VCC of the suprasellar arachnoid cyst. 

Cytoperitoneal shunting is a relatively straightforward procedure with the insertion of a catheter into the cyst. It provides a gradual reduction in the cyst cavity rather than a rapid shift in pressure. However, it places the patient at a life-long shunt dependence and the risk associated with it, including shunt malfunction, cystic hemorrhage, and/or infection. A neuro-endoscopic approach offers a minimally invasive option without any lasting hardware. It does require a higher technical demand and training due to the single instrument and proximity to crucial anatomical structures. There is an increased risk of subdural effusion; however, there is less risk of recurrence and infection. Finally, via either endoscopy or craniotomy, cyst resection has a higher risk of injuring the underlying structures and hemorrhage due to retraction and maneuvering of the cystic wall [9].

The efficacy and superiority of each method are still a topic of debate. Nevertheless, the presence of obstructive hydrocephalus is an indicator for immediate endoscopic fenestration over craniotomy or shunting. Patients who present with hydrocephalus have better outcomes with a neuroendoscopic approach. This approach has a low risk of surgical complications than craniotomy and shunting [7]. Analysis of 23 cases showed a good clinical-radiological outcome of 79% in craniotomy and cyst excision, 85.7% in shunts, and 90% after endoscopic fenestration [10]. Thus, endoscopic fenestration is superior over shunting and surgical excision in patients who present with hydrocephalus.

Within neuroendoscopic fenestration, various techniques have been analyzed to determine the efficacy and rate of both short-term and long-term complications. The fenestration can be performed only at the apical membrane, usually at the level of the foramen of Monro, between the ventricle and the cyst (ventriculocystostomy or VC), or to basilar fenestration toward the prepontine cistern called VCC [7]. Dual fenestration into the interventricular compartment and basal cisterna is superior to apical fenestration due to decreased risk of relapse of symptoms [11]. Moreover, Maher et al. has demonstrated that VCC has less risk of reoperation [12]. Furthermore, another study showed that 8% of patients who underwent VCC as the primary treatment required reoperation vs. sixteen percent who underwent VC as primary surgery required reoperation [12]. Finally, the radiological-clinical improvement rate was higher in VCC (100%) compared to 81.8% in VC [13]. This superiority can be attributed to the fact the apical fenestration can close due to the stretching of the third ventricle and eventual scarring. The basal opening's persistence allows for continued cyst decompression even if the superior fenestrate seals post-surgery [1].

## Conclusions

Suprasellar arachnoid cysts are cerebrospinal fluid dilations that cause obstructive hydrocephalus, ataxia, and other endocrinological disorders. The treatment requires communication of the cyst to the surrounding ventricles or cisterns. The current literature supports the superiority of the VCC over other interventions. Our case presentation highlights the neuroendoscopic VCC in a three-year-old patient and provides a step-by-step illustration of a safe, neuronavigation-assisted, non-cautery technique and the relevant third ventricular anatomy. 
